# Effect of Bentonite on the Electrical Properties of a Polylactide-Based Nanocomposite

**DOI:** 10.3390/polym16101372

**Published:** 2024-05-11

**Authors:** Jacek Fal, Katarzyna Bulanda, Mariusz Oleksy, Gaweł Żyła

**Affiliations:** 1 Department of Physics and Medical Engineering, Faculty of Mathematics and Applied Physics, Rzeszów University of Technology, 35-959 Rzeszów, Poland; gzyla@prz.edu.pl; 2 Department of Polymer Composites, Faculty of Chemistry, Rzeszów University of Technology, 35-959 Rzeszów, Poland; k.bulanda@prz.edu.pl (K.B.); molek@prz.edu.pl (M.O.)

**Keywords:** electrical properties, polylactide, bentonite, nanocomposite

## Abstract

In this paper, a novel polylactide-based nanocomposite with the addition of bentonite as a filler, Fusabond, and glycerine as a compatibilizer and plasticizer, were prepared and investigated. Four samples with different contents of bentonite (1, 5, 10, and 15 wt.%), as well as three samples without fillers, were prepared with an easily scalable method: melt blending. The electrical properties of all prepared samples were investigated with broadband dielectric spectroscopy in the frequency range between 0.1 Hz and 1 MHz. Measurements were conducted at nine temperatures between 293.15 and 333.15 K (20 to 60 °C) with steps of 5 K. It was found that the increase in the content of bentonite in polylactide has a significant effect on the electrical properties of the prepared nanocomposites.

## 1. Introduction

Since plastic was invented, it has become an integral part of our lives, with the industry bringing many benefits such as lightweight tools and packaging. Unfortunately, with the massive use of various types of synthetic materials, the resulting environmental risks have begun to be recognized. The main problem is the very long decomposition time of plastics from the petroleum industry and difficulties in recycling, resulting in their indiscriminate disposal in landfills and beyond [1,2,3]. A step toward solving this problem could be the use of non-petroleum-derived plastics. One such material is polylactide (PLA) which is a biodegradable and bio-based polymer that has gained significant attention in recent years as a more environmentally friendly substitute for conventional petroleum-derived materials [4].

PLA is derived from renewable resources such as corn starch or sugarcane, making it an eco-friendly choice for various applications [5,6]. However, its intrinsic brittleness, low thermal resistance, and limited processing versatility constrain its use [7]. To address these limitations, scientists have begun to study the effect of various additives on PLA as reinforcement, leading to the development of PLA nanocomposites [8,9]. Nanocomposites present a novel group of materials with mechanical, physical, and chemical properties that can be tailored for specific applications by reinforcement with various fillers and nanofillers. Nanocomposites are recognized as materials where nanoscale fillers are dispersed within a matrix. To consider the material a nanocomposite, at least one of the filler dimensions should be less than 100 nm. As fillers, various types of nanoparticles, nanotubes, or other types of nanostructures can be used [10,11,12].

One of the most intensively explored directions of nanocomposites research is improving their mechanical properties. Steps in this direction have been taken by Ibrahim et al., among others [13]. They prepared nanocomposites of polylactide with organoclay with a mass concentration between 2 and 10%. Their results showed that the nanofiller had a significant impact on the mechanical characteristics of the polylactide. At the highest measured nanofiller content (10 wt.%), it was discovered that Young’s modulus increased by 54% but tensile strength dropped by almost 50%. Mohapatra et al. [14] carried out a similar investigation on the impact of filler content (1–5 wt.%). For a filler concentration of 5 wt.%, they demonstrated an increase in the elasticity modulus and a reduction in elongation of nearly 70%. On the other hand, Coppola et al. [15] examined the mechanical characteristics of a nanocomposite made from a polylactide matrix and reinforced with layered silicates using incremental technology (3D printing). They succeeded in producing a nanocomposite material for 3D printing with an elasticity modulus that is 15% more than that of PLA. Petersson et al. [16] modified the polylactide with maleic anhydride and added fillers in the form of two types of layered silicates (bentonite and hectorite) to further enhance the mechanical properties of the polylactide. They achieved 63% improvement in the yield strength and 82% enhancement in tensile modulus with respect to pure PLA; however, the elongation at breaking point was decreased in comparison to pure PLA. More information concerning the mechanical properties of PLA-based nanocomposites can be found in a small number of recent [17] and previous review papers [7,18,19,20].

Besides the improvement in the mechanical properties of nanocomposites based on PLA, its thermal stability was also enhanced by introducing fillers and nanofillers into a polymer matrix. It was shown by Bandyopadhyay et al. [21] that the addition of fluorohectorite-modified silicate particles to the polylactide causes improvement in the thermal stability of the prepared nanocomposite. Similar conclusions were reached by Paul et al. [22] who produced nanocomposites based on a natural polylactide with modified montmorillonite (MMT). They discovered that the PLA matrix has the greatest thermal stability when the MMT concentration is up to 5 wt.%. The thermal stability of the investigated nanocomposites decreases as the MMT content rises. Additionally, it was demonstrated that the melting and glass transition temperatures are unaffected by the matrix’s inclusion of montmorillonite. Similar findings were made by Pluta et al. [23], who discovered that polylactide nanocomposites with montmorillonite contents up to 3 wt.% may be able to enhance their thermal properties.

It has also been shown that the modification of the nanocomposite preparation method can moderate their thermal properties. The thermal stability of nanocomposites made by pressing alternating layers of polylactide and montmorillonite was studied by Wu et al. [24]. According to their findings, the prepared nanocomposites degraded more slowly than traditional polylactides. Additionally, they discovered that as the concentration of montmorillonite in the polylactide matrix increased, the melting temperature decreased. Thellen et al. [25] also studied the effect of the preparation method of nanocomposites on their thermal properties. They prepared polylactide nanocomposite with 5 wt.% of montmorillonite using different screw speed values during fabrication. They show that regardless of the screw speed, a 9 K rise in the disintegration temperature of the nanocomposites tested occurred while the melting temperature remained unchanged. The favorable effect of layered silicates on the thermal stability of nanocomposites based on polylactide matrix has also been confirmed many times [26,27,28,29,30]. The possibility of using nanocomposites in ionizing radiation shielding has been intensively studied in recent years. Polymer-based nanocomposites could be particularly interesting in medicine, where X-rays are a common tool in diagnostics as it was described in recent review papers [31,32]. On the other hand, Sobczak and Żyła summarized the latest developments in experimental and simulation studies on shielding properties of various composites, including polymer-, glass- and cement-based materials. They also described the possibilities of using these materials in the nuclear energy sector [33]. The nanocomposites exhibit some superior properties to the classic radiation shielding materials (especially lead), which, besides its lower toxicity, is its flexibility and easy formulation [34,35,36]. Hence, this area seems to be of significant interest.

Literature studies show that introduction fillers and nanofillers can also affect the electrical properties of nanocomposites. Despite the fact that most of these studies concern the use of highly conductive nanoparticles (NP), based on carbon [37,38,39,40,41,42] or silver [43,44], attempts are being made to use less obvious fillers. Mujeeb et al. [45] used a solution casting method of prepared nanocomposites based on polylactide and aluminum oxide nanoparticles. Their studies showed a 35–45% reduction in resistivity of PLA. In turn, Fal et al. [46] investigated the effect of silicon oxide–lignin nanoparticles on the polylactide matrix. They prepared four samples with mass concentrations between 1 and 15 wt.% using melt blending method. The obtained nanocomposites were characterized by increased electrical conductivity, and the highest enhancement was observed for 10 and 15 wt.%, which was several orders of magnitude. The effect of the combination of two different fillers was also investigated by Santangelo et al. [47]. They used carbon nanotubes and clay as a filler and achieved a 6–9 order of magnitude increase in electrical conductivity, depending on used concentration of fillers. Similar studies with clay and carbon nanotubes were also conducted by Gorrasi et al. [48].

The effect of montmorillonite on the dielectric properties of polylactide was studied by Pluta et al. [23]. They concluded that dielectric spectra of all nanocomposites are influenced by direct current (DC) conductivity, Maxwell/Wagner/Sillars polarization, and electrode polarization, especially at high temperatures. Dielectric properties of polylactide-based nanocomposites were also studied by Wu et al. [49] in the context of using TiO_2_-decorated carbon nanotubes to improve its dielectric constant. They achieved a dielectric constant 8.3 times higher than that in the case of pure PLA. The effect of nanoclay on the electrical properties of polylactide nanocomposite after the recycling process was studied by Salah et al. [50]. Their findings show a significant enhancement in conductivity for recycled nanocomposite and a marginal impact on the dielectric constant.

In this study, to expand the possibilities of PLA applications, the effect of bentonite on the electrical properties of polylactide-based nanocomposites was investigated. For this purpose, bentonite as a novel filler was introduced to the PLA matrix with four mass concentrations (1, 5, 10, and 15 wt.%) to improve the electrical conductivity of polylactide matrix and modify their permittivity. For comparison, pristine PLA and two additional samples without fillers were also studied. The presented studies provide insight into the electrical properties of bentonite–polylactide nanocomposite, which are often overlooked in comparison with the mechanical properties of nanocomposites.

## 2. Materials and Methods

### 2.1. Materials

For the preparation of nanocomposites, the natural and colorless polylactide (Proprox, Chwaszczyno, Poland) was used as the main component of the matrix. PLA in the form of a 3D printing filament was mixed with anhydrous glycerine (G) (Chempur, Piekary Śląskie, Poland) and the maleic anhydride grafted polyethylene (F) (Fusabond E226, DuPont, Wilmington, DE, USA) as a plasticizer and a compatibilizer, respectively. As a nanofiller, an unmodified bentonite (BE) (Zębiec, Zakłady Gurniczo-Metalowe S.A., Zębiec, Poland) was applied. According to the manufacturer, the filler contains more than 75% montmorillonite, a maximum of 5% carbonate, and less than 13% water, and the remaining 7% is composed of smectite, calcite, dolomite, feldspar, kaolinite, and quartz.

### 2.2. Sample Preparation

All samples of nanocomposites and the reference PLA were manufactured using the melt blending method. The ratio of ingredients (G and F) used was chosen based on previous studies and the team’s years of experience [51]. First, using an analytical balance, the proper proportions of each component were weighed (Pioneer Semi-Micro PX225DM, OHAUS Corporation, Parsippany, NJ, USA) and then mechanically mixed. The pre-mixture was then placed in a co-rotating twin-screw extruder (HAAKE MiniLab II, Thermo Fisher Scientific, Karlsruche, Germany) to extrude the nanocomposite thread. The melt-blending process was carried out at 463.15 K (190 °C) with a screw speed of 50 rpm. In the next step, the obtained nanocomposite threads were granulated and placed in an injection molding HAAKE MiniJet II (Thermo Fisher Scientific, Karlsruche, Germany) to prepare bar-shaped samples (10 × 60 × 1 mm^3^). The final shape of samples (20 mm in diameter) was obtained using a cylindrical knife and heated press. The list of prepared samples with specific proportions of ingredients, expressed as a mass concentration, $\varphi_{m}$, and used labels is presented in Table 1.

### 2.3. Characterization Methods

The characterization of the fracture surface of prepared nanocomposites was performed with a scanning electron microscope (SEM) (Hitachi S-3400N, Hitachi Ltd., Tokyo, Japan). SEM observation was carried out using a backscattered electron detector (BSE) and a 5 kV accelerating voltage in both high- and low-vacuum modes (LV-50 Pa).

The electrical properties of BE-PLA nanocomposites were investigated using broadband dielectric spectroscopy (Concept 80 System, Novocontrol GmbH, Montabaur, Germany). A frequency range of 1 MHz to 0.1 Hz was applied to obtain frequency characteristics of nanocomposites at temperatures between 293.15 and 333.15 K (20 and 60 °C) every 5 K.

Values of DC electrical conductivity were obtained based on the fragments of AC conductivity spectra, where they were frequency-independent (plateau). If a plateau was not observed, the value of DC electrical conductivity was taken as a value of $\sigma_{AC}$ obtained at the lowest tested frequency (0.1 Hz). Detailed information regarding the measuring device, procedure, and standard deviation of measurements (less than 6%), which was determined based on 10 measurements of glycerin, can be found in previously published papers [38,46].

## 3. Results

### 3.1. SEM Investigation

**Figure 1:**
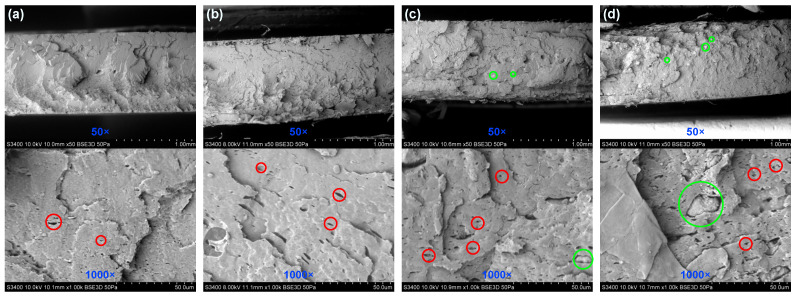
SEM images of a fractured surface of the BE-PLA-FG nanocomposite: (**a**) 1 wt.%, (**b**) 5 wt.%, (**c**) 10 wt.%, (**d**) 15 wt.%. Red circles show examples of voids, while green circles show examples of possible agglomerates.

The scanning electron microscopy of the fractured surface of BE-PLA-FG nanocomposites is presented in Figure 1. The observed fractures are characterized by many irregular planes of cracks, and no significant difference in their number and size can be seen between different concentrations of nanofiller. Additionally, numerous voids on the surface can be noted in the case of each sample (see the red circles in Figure 1), which might be related to the presence of glycerine and Fusabond in the matrix. Moreover, in the case of BE-PLA-FG-1 and BE-PLA-FG-5 scanning electron microscope images confirm the good dispersity of filler in the matrix. There were no visible agglomerates on the surface, while for BE-PLA-FG-10 and BE-PLA-FG-15 nanocomposites, some bentonite agglomerates could be captured (see green circles in Figure 1). SEM images of samples without fillers and their analysis can be found elsewhere [46].

### 3.2. Electrical Properties

The electric polarizability of a dielectric material is measured using its complex dielectric permittivity, $\varepsilon^{*}$ which consists of two components. The real part of the complex permittivity, $\varepsilon^{\prime }$, also named the dielectric constant, reflects the material’s ability to store electrical energy. On the other hand, the imaginary part of complex permittivity, $\varepsilon^{\prime \prime }$, quantifies the energy dissipation within the material under an external alternating electric field; for this reason, it is also called the dielectric loss.

**Figure 2:**
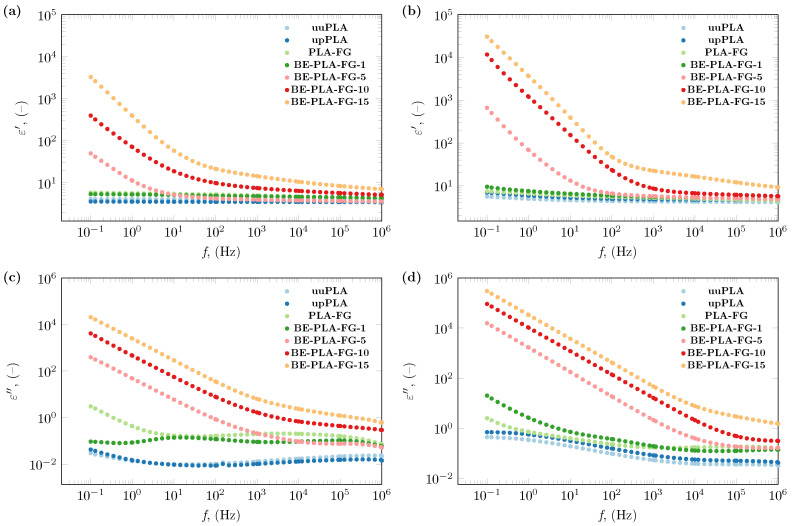
The dielectric constant of the BE-PLA-FG nanocomposite at (**a**) 293.15 K (20 °C) and (**b**) 333.15 K (60 °C), and dielectric loss at (**c**) 293.15 K (20 °C) and (**d**) 333.15 K (60 °C).

The dielectric behavior of BE-PLA-FG nanocomposites and three samples without the addition of nanofillers (uuPLA, upPLA, PLA-FG) are presented in Figure 2. The dielectric constant and dielectric loss of all studied samples were investigated in terms of the wide frequency range, and temperature. The representative results of these studies are presented in Figure 2, where the real and imaginary parts of permittivity spectra at 293.15 and 333.15 K (20 and 60 °C) can be observed. As it can be seen, $\varepsilon^{\prime }$ (Figure 2a,b) of samples without the addition of bentonite (upPLA, PLA-FG) remain almost insensitive to frequency changes, and the shape of their spectra is close to pristine PLA (uuPLA), as reported in [38,46,52]. The increase in temperature causes a slight increase in values of the dielectric constant (Figure 2a,b). On the other hand, there are visible differences in the dielectric loss of PLA-FG and polylactide without any additions (uuPLA and upPLA) (Figure 2c), which decreases with increasing temperature (Figure 2d).

The impact of bentonite load in the polylactide matrix is visible from the lowest tested concentration. Generally, an increase in the content of bentonite causes an increase in both the dielectric constant and the loss at each investigated temperature and throughout the tested frequency range. There is only one exception in the case of BE-PLA-FG-1, where real and imaginary parts of permittivity are lower or at a level akin to PLA-FG samples (especially in the range of lower frequencies) but still higher than pristine (uuPLA) and processed PLA (upPLA).

**Figure 3:**
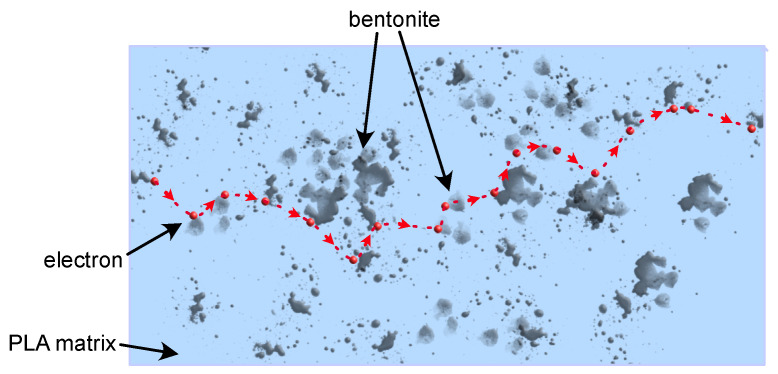
A diagram of the phenomenon of electrical conduction by tunneling is marked on the nanocomposite cross-section. The light blue color indicates the PLA matrix, the gray non-regular elements represent dispersed particles, the red dots show electrons, and the red dashed line shows the possible tunneling path of the charge carrier through the volume of the nanocomposite.

The effect of nanofiller (BE) is noticeable in the entire range of investigated frequencies. An increase in the load of bentonite causes an increase in values of both real and imaginary parts of permittivity, and the most significant changes introduced by the addition of bentonite particles can be observed in lower frequencies (Figure 2). An increase in dielectric constant and dielectric loss values is observable with decreasing frequency. A particularly noticeable increase in the value of the dielectric constant is observed below 100 Hz, which can be related to the creation of a tunneling conduction path through the sample. According to a theory presented by Wang et al. [53], increasing the amount of filler in the sample results in a reduction in the average distance between the nanoparticles, which may encourage efficient electron tunneling. Nan and other researchers assumed the same or similar conclusions may be true [54,55,56]. A simplified graphical representation of this phenomenon is shown in Figure 3.

Additionally, in the low-frequency range, it was observed that the Maxwell/Wagner/Sillars effect occurs on the interfacial boundaries, which is most visible for the BE-PLA-FG-1 sample at 3 Hz. In the case of samples with a higher content of bentonite (5, 10, and 15 wt.%), this effect is masked by the conductivity component. Similar observations have been made for nanocomposites based on polylactide and a mixture of graphite and diamond [38].

**Figure 4:**
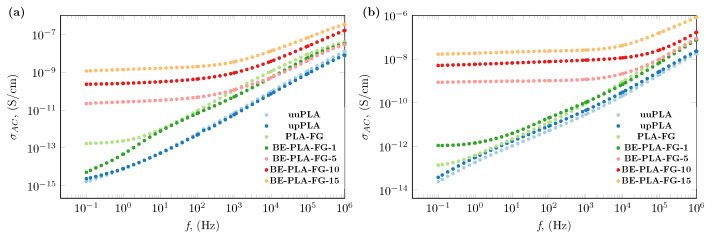
AC electrical conductivity, $\sigma_{AC}$ of BE-PLA-FG nanocomposite with different mass concentration, $\varphi_{m}$ of bentonite (BE) at (**a**) 293.15 K (20 °C) and (**b**) 333.15 K (60 °C).

The addition of bentonite to polylactide has also impacted its AC electrical conductivity. Evidence of these is presented in Figure 4, where the conductivity spectra of BE-PLA-FG nanocomposites and three unfilled samples at two representative temperatures 293.15 K (20 °C) and 333.15 K (60 °C) were presented. As was previously stated, the injection process of pristine PLA does not significantly affect its electrical conductivity, especially at lower temperatures of 293.15 K (20 °C, as shown in Figure 4a). Only at higher temperatures of 333.15 K (60 °C, as shown in Figure 4b), can slightly higher values of electrical conductivity be noted in the entire frequency range. This slight increase in the value of σ was stamped out by the rise in lactic acid content that resulted from hydrolysis and degradation in higher temperatures. Meanwhile, the addition of glycerine and Fusabond to the polylactide resulted in an increase in electrical conductivity throughout the whole tested frequency range, which may be due to the concentration of hydronium cations increased overall [38]. Additionally, unfilled samples exhibit strong frequency dependences of AC conductivity that are observed in the case of electroinsulating materials [49]. In contrast, samples containing a load of bentonite show regions where values of electrical conductivity of nanocomposites are almost unaffected by frequency changes (plateau). This behavior is observed in the low-frequency range, and the span of its occurrence increases as the filler content increases. After the plateau, a rapid increase in electrical conductivity is observed with increasing frequency. Such behavior is in agreement with universal power law expressed as follows [57]

(1) $$\sigma =\sigma_{DC}+A\omega^{n},$$

where *A* and *n* are numerical factors and $\sigma_{DC}$ is DC electrical conductivity, while ω means angular frequency. Similar electrical conductivity spectra were previously observed for various types of nanocomposites filled with different types of nanoparticles [38,46,58,59,60,61].

**Figure 5:**
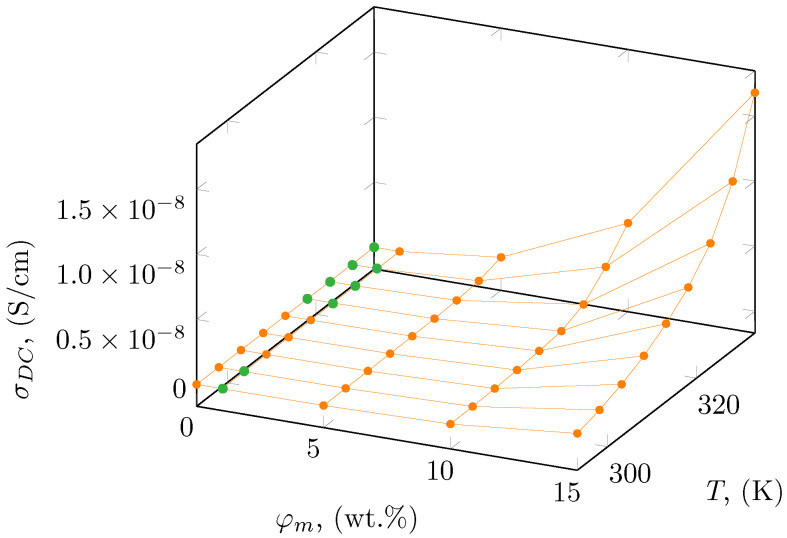
Electrical conductivity, $\sigma_{DC}$ of BE-PLA-FG nanocomposites as a function of mass concentration, $\varphi_{m}$, and temperature, where 0 wt.% corresponds to the PLA-FG sample. Green points correspond to values obtained at 0.1 Hz, and orange points correspond to values obtained based on the plateau.

Based on the observed plateaus, the DC electrical conductivity was designated and is summarized in Table 2, as well as presented in Figure 5 as a function of temperature and mass concentration. In the case where a plateaus region has not been observed, the value of DC conductivity was taken as a value of AC conductivity at the lowest measured frequency (0.1 Hz). The obtained results show a moderate increase in electrical conductivity for a mass concentration below 5 wt.% with increasing temperature, while above 10 wt.% enhancement in electrical conductivity with temperature is significant. A similar enhancement was obtained by Pluta et al. [52] for a polylactide-based nanocomposite with the addition of organically treated montmorillonite. They presented the electrical conductivity as 3.25 × 10^−10^ and 1.3 × 10^−9^ μS/cm for 3 and 10 wt.%, respectively, of montmorillonite in the polylactide matrix, which is similar to the results presented for BE-PLA-10 and BE-PLA-15.

## 4. Conclusions

Nanocomposites based on polylactide with bentonite as a filler were prepared in four mass concentrations (1, 5, 10, and 15 wt.%). Additionally, three samples without any filler were also prepared and investigated. Fractured surfaces of all samples were studied with SEM, and good dispersity was confirmed for samples with a load of bentonite below 5%. Above this content, some agglomerates were observed, which do not have a negative effect on the electrical conductivity of these samples. Investigations of the electrical properties of all samples were carried out in a temperature range between 293.15 and 333.15 K (20 and 60 °C) and a frequency span from 1 MHz to 0.1 Hz. Based on the results obtained, DC conductivity was designated, and it was stated that an increase in the load of bentonite in the polylactide matrix causes an increase in electrical conductivity in the whole investigated temperature range. The highest value of electrical conductivity was observed for the highest content of filler (15 wt.%) at the highest tested temperature, and it was 1.68 × 10^−8^ S/cm.

## Figures and Tables

**Table 1:** Labels and ingredient ratios (in mass concentration, $\varphi_{m}$, nanocomposite) samples that have been manufactured.

Full Name	Label	BE	F	G	PLA
unfilled and unprocessed PLA	uuPLA	-	-	-	1
unfilled and processed PLA	upPLA	-	-	-	1
mixture of PLA, glycerine, Fusabond	PLA-FG	-	0.5	20	79.5
bentonite (1 wt.%) PLA	BE-PLA-FG-1	1	0.5	20	78.5
bentonite (5 wt.%) PLA	BE-PLA-FG-5	5	0.5	20	74.5
bentonite (10 wt.%) PLA	BE-PLA-FG-10	10	0.5	20	69.5
bentonite (15 wt.%) PLA	BE-PLA-FG-15	15	0.5	20	64.5
BE—bentonite, F—maleic anhydride grafted polyethylene, G—glycerine, PLA—polylactide.					

**Table 2:** Values of DC electrical conductivity designated based on AC electrical conductivity spectra at low frequencies for BE-PLA-FG nanocomposites at various temperatures. Data highlighted in green correspond to values obtained at 0.1 Hz.

T, K	$\sigma_{\mathrm{DC}}$, (S/cm)						
	**uuPLA**	**upPLA**	**PLA-FG**	**BE-PLA-FG-1**	**BE-PLA-FG-5**	**BE-PLA-FG-10**	**BE-PLA-FG-15**
293.15	1.63 × $10^{-15}$	2.34 × $10^{-15}$	1.69 × $10^{-13}$	5.16 × $10^{-15}$	2.18 × $10^{-11}$	2.32 × $10^{-10}$	1.15 × $10^{-9}$
298.15	1.91 × $10^{-15}$	2.45 × $10^{-15}$	1.62 × $10^{-13}$	2.31 × $10^{-14}$	2.52 × $10^{-11}$	2.62 × $10^{-10}$	1.64 × $10^{-9}$
303.15	2.25 × $10^{-15}$	2.66 × $10^{-15}$	1.25 × $10^{-13}$	3.70 × $10^{-14}$	3.49 × $10^{-11}$	3.24 × $10^{-10}$	2.29 × $10^{-9}$
308.15	2.63 × $10^{-15}$	3.08 × $10^{-15}$	1.17 × $10^{-13}$	4.07 × $10^{-14}$	5.03 × $10^{-11}$	4.27 × $10^{-10}$	3.16 × $10^{-9}$
313.15	3.22 × $10^{-15}$	3.74 × $10^{-15}$	9.66 × $10^{-14}$	2.98 × $10^{-14}$	7.00 × $10^{-11}$	5.77 × $10^{-10}$	4.31 × $10^{-9}$
	**uuPLA**	**upPLA**	**PLA-FG**	**BE-PLA-FG-1**	**BE-PLA-FG-5**	**BE-PLA-FG-10**	**BE-PLA-FG-15**
318.15	4.16 × $10^{-15}$	5.15 × $10^{-15}$	4.45 × $10^{-14}$	2.82 × $10^{-14}$	1.01 × $10^{-10}$	7.85 × $10^{-10}$	5.78 × $10^{-9}$
323.15	6.31 × $10^{-15}$	8.75 × $10^{-15}$	6.39 × $10^{-14}$	3.71 × $10^{-14}$	1.91 × $10^{-10}$	1.52 × $10^{-9}$	7.86 × $10^{-9}$
328.15	1.46 × $10^{-14}$	2.36 × $10^{-14}$	1.33 × $10^{-13}$	4.37 × $10^{-14}$	3.72 × $10^{-10}$	3.09 × $10^{-9}$	1.13 × $10^{-8}$
333.15	2.43 × $10^{-14}$	3.84 × $10^{-14}$	1.39 × $10^{-13}$	1.11 × $10^{-12}$	8.69 × $10^{-10}$	5.13 × $10^{-9}$	1.68 × $10^{-8}$

## Data Availability

Data are contained within the article.
